# Knowledge, Attitude and Practice of Brazilian Obstetricians Regarding Episiotomy

**DOI:** 10.1055/s-0039-3400314

**Published:** 2019-11

**Authors:** Carolina Maria Pires Cunha, Leila Katz, Andrea Lemos, Melania Maria Amorim

**Affiliations:** 1 Instituto de Medicina Integral Professor Fernando Figueira, Recife, PE, Brazil; 2 Universidade Federal de Pernambuco, Recife, PE, Brazil

**Keywords:** episiotomy, health knowledge, attitudes in health, practices in health, obstetrics, delivery, perineum, episiotomia, conhecimentos em saúde, atitudes em saúde, práticas em saúde, parto, obstetrícia, períneo

## Abstract

**Objective** To determine the prevalence of episiotomy and the factors associated with the knowledge, attitude and practice (KAP) of Brazilian obstetricians in relation to this procedure.

**Methods** A KAP survey was conducted with obstetricians working in Brazil. An electronic form containing structured questions previously evaluated using the Delphi method was created in Google Docs and sent by e-mail. A multivariate logistic regression was performed to determine the principal factors associated with adequate KAP. For each dependent variable (knowledge, attitude and practice) coded as adequate (1 = yes; 0 = no), a multiple logistic regression model was developed. Binary codes (1 = yes and 0 = no) were assigned to every independent or predictor variables. Prevalence ratios (PRs) and their respective 95% confidence intervals (95%CIs) were calculated as measures of relative risk, at a significance level of 5%.

**Results** Out of the 13 thousand physicians contacted, 1,163 replied, and 50 respondents were excluded. The mean episiotomy rate reported was of 42%. Knowledge was determined as adequate in 44.5% of the cases, attitude, in 10.9%, and practice, in 26.8% of the cases.

**Conclusion** Most respondents had inadequate knowledge, attitudes and practices regarding episiotomy. Although some factors such as age, teaching, working in the public sector and attending congresses improved knowledge, attitude and practice, we must recognize that episiotomy rates remain well above what would be considered ideal. Adequate knowledge is more prevalent than adequate attitude or practice, indicating that improving knowledge is crucial but insufficient to change the outlook of episiotomies in Brazil.

## Resumo

**Objetivo** Determinar o nível e os fatores associados a conhecimento, atitude e prática (CAP) dos obstetras brasileiros em relação à episiotomia.

**Métodos** Foi realizado um estudo de corte transversal do tipo inquérito CAP com obstetras atuantes no território brasileiro. Foi criado um formulário eletrônico com perguntas estruturadas e previamente avaliadas pelo método Delphi, que foi enviado por e-mail pelo sistema Google Docs. Foi realizada uma análise multivariada de regressão logística múltipla para determinação dos principais fatores associados ao conhecimento, à atitude e à prática adequados. Para cada variável dependente (conhecimento, atitude e prática) codificada como adequada (1 = sim; 0 = não) foi construído um modelo de regressão logística múltipla. Todas as variáveis independentes ou preditivas foram codificadas binariamente (1 = sim; 0 = não). As razões de prevalência (RPs) e seus respectivos intervalos de confiança de 95% (ICs95%) foram calculados como medidas do risco relativo, com um nível de significância de 5%.

**Resultados** Dos 13 mil obstetras contatados, foram obtidas 1.163 respostas, sendo 50 participantes excluídos. A média da taxa de episiotomia relatada foi de 42%. Verificou-se que 44,5% dos médicos tinham conhecimentos adequados, 10,9% tinham atitudes adequadas e 26,8% tinham práticas adequadas.

**Conclusão** A maioria dos participantes tinha conhecimento, atitudes e práticas inadequadas em relação à episiotomia. Embora alguns fatores como idade, ensino, trabalho no setor público e participação em congressos melhorem o conhecimento, a atitude e a prática, é preciso reconhecer que as taxas de episiotomia permanecem bem acima do que se considera ideal. O conhecimento adequado é mais prevalente do que a atitude ou a prática adequadas, indicando que a melhora do conhecimento é crucial, mas insuficiente para mudar o panorama das episiotomias neste país.

## Introduction

Currently available scientific evidence corroborates the selective use of episiotomy.^1^ ^2^ In its last guideline,^3^ the World Health Organization (WHO) recommends against routine/liberal episiotomy, and acknowledges that there is no evidence to support any indication of episiotomy in modern obstetrics; therefore an “ideal” rate of episiotomy persists to be established. The American College of Obstetricians and Gynecologists (ACOG) established in 2006 and corroborated in 2018 that episiotomy should be restricted, and that physicians should use their clinical judgment to decide when the procedure is necessary,^4^ since there is no clinical evidence supporting any indication for episiotomy.^1^ ^4^ The well-documented risks of routine episiotomy include severe perineal trauma, perineal pain and healing complications.^1^

Despite the worldwide trend toward a reduction in episiotomy rates, the actual performance of this procedure varies considerably from region to region.^5^ ^6^ A retrospective study conducted in Israel reported a decline in the rate of episiotomy from more than 30% in the 1990s to less than 5% in 2010.^7^ In hospital deliveries in Brazil, episiotomy rates vary considerably; however, the practice remains common. Episiotomy rates around the country declined from ∼ 94% in 2000^8^ to 76% in 2006^9^ and 54% in 2014.^10^ Nevertheless, this rate is still high, mainly when compared with other countries such as the United States (24.5%),^11^ France (13.3%)^12^ and the Netherlands (10.8%).^13^

Various interventions have been proposed in an attempt to reduce episiotomy rates.^14^ Disseminating evidence and improving knowledge on episiotomy is important. In the United States, following the publication of a systematic review^2^ and the dissemination of the 2006 ACOG guidelines, a significant decline occurred in the performance of episiotomies, with rates expected to keep on falling.^14^ Even so, to ensure that existing knowledge is translated efficiently into practice, not only the healthcare professionals' opinion and their level of knowledge has to be investigated, but also the effect of their obstetric training, in order to increase our understanding of what the professionals know and what they practice.^15^

The knowledge, attitude and practice (KAP) survey is a technique used to measure what individuals know, what they believe, and how they act in certain situations. These surveys contribute to improve health education because educators may diagnose their students prior to implementing strategies aimed at changing inadequate practices.^16^

The objective of the present study was to determine the level of knowledge, and the attitudes and practices of obstetricians living in Brazil regarding episiotomy and the factors associated with adequate KAP.

## Methods

### Study Design and Patient Recruitment

The present KAP study included obstetricians living in Brazil and affiliated to the Brazilian Federation of Gynecology and Obstetrics Associations (Federação Brasileira das Associações de Ginecologia e Obstetrícia, FEBRASGO, in Portuguese), the largest medical association in the field of Gynecology and Obstetrics in Brazil. The review board of our institution approved the study prior to its beginning under reference number 23410613.9.0000.520.

### Outcomes (KAP and Episiotomy)

An invitation to participate in the survey was sent by e-mail from the FEBRASGO headquarters to all the members included in its electronic database (more than 13 thousand doctors). A structured self-administered questionnaire prepared by specialists using the Delphi method was used to collect the data.^17^ The final questionnaire contained only pre-coded questions, with the participant being asked to check the answers they judged pertinent. The questionnaire was answered anonymously, thus guaranteeing confidentiality. Prior to filling out the form, the participants signed an electronic informed consent form.

The possible answers regarding questions on knowledge were “true,” “false” or “I don't know.” As for attitude, a Likert-type scale was used to grade five response categories:^18^ “I agree,” “I completely agree,” “I disagree,” “I completely disagree,” and “I don't know.” Regarding practice, the possible polychotomous answers were “always,” “sometimes,” “seldom,” and “never.” In addition, the obstetricians were asked to inform their episiotomy rate over the previous year.

For the data analysis, knowledge was considered adequate when the participant answered “true” regarding all true statements, or “false” in the case of false statements. Knowledge was considered inadequate when the answer for true statements was “false” or “I don't know,” or when the answer for false statements was “true” or “I don't know.” The statements used to evaluate knowledge were based on a Cochrane systematic review,^1^ and on the ACOG^4^ and the WHO guidelines.^3^

Attitude was considered adequate when the answer was “I agree” or “I completely agree” in the case of true statements, and “I disagree” or “I completely disagree” for false statements. Attitude was considered inadequate when the answer to a true statement was “I disagree,” “I completely disagree,” or “I don't know,” or when the answer to a false statement was “I agree,” “I completely agree” or “I don't know.” All of the questions on attitude were related to the performance of episiotomy and its consequences to the woman, also taking the best currently available scientific evidence into consideration.^1^ ^2^

With respect to practice, the episiotomy rate reported by the obstetrician (the percentage of episiotomies performed in the previous year considering the number of deliveries performed) was analyzed. Rates ≤ 20% of deliveries were considered adequate. This figure was compared with the participant's answer to the question in which his/her performance of episiotomy was evaluated as “always,” “sometimes,” “seldom” or “never.”

For each domain of the questionnaire, a score that ranged from 0 to 10 was calculated, with 10 being the highest score when all of the questions were answered correctly. The cut-off point was established as a minimum of 70% of correct answers.

Both the electronic questionnaire and the informed consent form were created in Google Docs. An e-mail was sent to all of the physicians in the FEBRASGO database. A further two identical emails were sent in an attempt to reach a greater number of potential respondents.

## Statistical Analysis

The results were exported from Google Docs to an Excel (Microsoft Office 2007, Microsoft Corp., Redmond, WA, US) database, and then analyzed using the Epi Info software, version 7.1.5, which is free and available for download on the internet. The characteristics of the sample population were analyzed using measures of central tendency and dispersion for the numerical variables, and distribution of frequency for the categorical variables. The answers provided in the KAP domains were analyzed to determine the frequency of those classified as adequate, and the episiotomy rates were calculated.

Contingency tables were developed to determine the association between the independent (predictive factors) and the dependent variables, that is, KAP in relation to episiotomy (the outcomes). A binary code was assigned to every dependent variable: adequate (yes) or inadequate (no). The significance level was set at 5%, and all *p*-values were two-tailed.

Prevalence ratios (PRs) and their respective 95% confidence intervals (95%CI) were calculated as measures of relative risk, with the significance level established at 5%. The standard risk of 1.0 was defined as the reference category.

Next, a multivariate logistic regression was performed to determine the principal factors associated with adequate KAP. For each dependent variable coded as adequate (1 = yes; 0 = no), a multiple logistic regression model was developed. Binary codes (1 = yes; 0 = no) were assigned to every independent or predictor variable.

In each model, the independent variables associated with the outcome at a 20% significance level were selected, and the first regression was then performed. Then, the variables no longer associated with the outcome were progressively removed from the model until a final model was obtained, which included only the variables associated with the outcome at a 5% significance level.

## Results

Of the 13 thousand gynecologists and obstetricians affiliated with FEBRASGO, 1,163 (9%) filled out and returned the questionnaire; however, 50 respondents were excluded because they did not practice obstetrics, leaving 1,113 filled-out questionnaires. In relation to the respondents' sociodemographic characteristics, there were slightly more women (53.6%) than men. The mean age was 44.3 ± 12.1 (standard deviation, SD) years, with 42.7% younger than 40 years of age. There was a predominance of participants from the Southeastern (48.8%) and Southern (20.3%) regions of Brazil. The majority (52.8%) worked in one of the state capital cities, both in the public and private sectors (57%). Most of the participants (69.5%) were specialists. Time since graduation was ≤ 10 years for 36.9%, between 10 and 25 years for 37.2%, and > 25 years for 25.9% of the respondents. Most had graduated from federal universities (51.6%). Slightly more than half (55%) did not teach, while 97.5% reported attending congresses, and 91.5% stated that they accessed electronic health databases.

Knowledge was found to be adequate among 44.5% of the physicians; attitude, among 10.9%, and practice, among 26.8%. Adequate knowledge was associated with: age < 40 years (PR: 1.2; 95%CI: 1.0–1.4); teaching (1.3; 1.1–1.5); accessing health databases (1.3; 0.9–1.7); time since graduation ≤ 10 years (1.3; 1.1–1.6); having a PhD (1.3; 0.9–1.9); working in the public sector (1.6; 1.3–2.0); and working in a state capital city (1.1; 0.9–1.3). The other variables (gender and attending congresses) were not significantly associated with knowledge (Table 1).

**Table 1:** Factors associated with adequate knowledge of Brazilian obstetricians regarding episiotomy

Variables	Adequate		Inadequate		PR	95%CI	*p*-value
	N	%	N	%			
**Age**	42.4	11.5	45.8	12.7			< 0.001
**Age < 40 years**							
Yes	249.0	50.0	259.0	50.0	1.25	1.09–1.42	< 0.001
No	246.0	40.0	369.0	60.0	1.00		
**Gender**							
Female	269.0	45.1	328.0	54.9	1.02	0.90–1.17	0.33
Male	226.0	43.8	290.0	56.2	1.00		
**Participation in congresses**							
Yes	485.0	44.7	600.0	55.3	1.25	0.75–2.06	0.17
No	10.0	35.7	18.0	64.3	1.00		
**Access to databases**							
Yes	462.0	45.4	556.0	54.6	1.30	0.98–1.73	0.02
No	33.0	34.7	62.0	65.3	1.00		
**Teaching**							
Yes	259.0	51.7	242.0	48.3	1.34	1.17–1.52	< 0.001
No	236.0	38.6	376.0	61.4	1.00		
**Time since graduation**							
≤ 10 years	189.0	49.6	192.0	50.4	1.36	1.14–1.61	< 0.001
10–25 years	179.0	46.7	204.0	53.3	1.28	1.07–1.63	0.004
> 25 years	127.0	36.4	222.0	63.6	1.00		
**Highest degree**							
Specialist	329.0	42.5	445.0	57.5	1.06	0.78–1.44	0.69
Master's degree	78.0	48.1	84.0	51.9	1.02	0.85–1.68	0.26
Doctor of Philosophy (PhD)	62.0	55.4	50.0	44.6	1.38	0.98–1.94	0.04
Doctor of Medicine (MD)	26.0	40.0	39.0	60.0	1.00		
**Place of work**							
State capital city	266.0	45.2	322.0	54.8	1.12	0.97–1.30	0.11
State capital and other cities	58.0	58.0	42.0	42.0	1.44	1.17–1.76	0.001
Outside state capital city	171.0	40.2	254.0	59.8	1.00		
**Employment sector**							
Public	130.0	54.4	109.0	45.6	1.65	1.33–2.04	< 0.001
Public and private	286.0	45.1	348.0	54.9	1.37	1.12–1.67	0.001
Private	79.0	32.9	161.0	67.1	1.00		

Abbreviations: 95%CI, 95% confidence interval; PR, prevalence ratio; SD, standard deviation.

Adequate attitude was associated with: being female (PR: 1.8; 95%CI: 1.2–2.6); age < 40 years (2.2; 1.5–3.1); time since graduation ≤ 10 years (2.2; 1.4–3.6); having a master's degree (9.2; 1.2–66.9); working in the public sector (2.5; 1.4–4.6); attending congresses (0.4; 0.2–1.0); teaching (1.5; 1.0–2.1); and having adequate knowledge (2.9; 2.03–4.27) (Table 2).

**Table 2:** Factors associated with adequate attitude in Brazilian obstetricians regarding episiotomy

Variables	Adequate		Inadequate		PR	95%CI	*p*-value
	N	%	N	%			
**Age**	39.3	9.7	44.9	12.3			< 0.001
**Age < 40 years**							
Yes	78.0	15.6	420.0	84.3	2.24	1.57–3.18	< 0.001
No	43.0	6.9	572.0	93.1	1.00		
**Gender**							
Female	82.0	13.7	515.0	86.3	1.81	1.26–2.61	< 0.001
Male	39.0	7.6	477.0	92.4	1.00		
**Participation in congresses**							
Yes	115.0	10.6	970.0	89.4	0.49	0.23–1.02	0.04
No	6.0	21.4	22.0	78.6	1.00		
**Access to databases**							
Yes	110.0	10.8	908.0	89.2	0.93	0.52–1.67	0.39
No	11.0	11.6	84.0	88.4	1.00		
**Teaching**							
Yes	67.0	13.4	434.0	86.6	1.51	1.08–2.12	0.007
No	54.0	8.8	558.0	91.2	1.00		
**Time since graduation**							
≤ 10 years	57.0	15.0	324.0	85.0	2.27	1.43–3.60	< 0.001
10–25 years	41.0	10.7	342.0	89.3	1.62	1.00–2.93	0.04
> 25 years	23.0	6.6	326.0	93.4	1.00		
**Highest degree**							
Specialist	86.0	11.1	688.0	88.9	7.22	1.02–51.01	0.005*
Master's degree	23.0	14.2	139.0	85.8	9.22	1.27–66.93	0.005*
Doctor of Philosophy (PhD)	11.0	9.8	101.0	90.2	6.38	0.84–48.32	0.002*
Doctor of Medicine (MD)	1.0	1.5	64.0	98.5	1.00		
**Place of work**							
State capital city	69.0	11.7	519.0	88.3	1.34	0.92–1.96	0.12
State capital and other cities	15.0	15.0	85.0	85.0	1.72	0.98–3.01	0.05
Outside state capital city	37.0	8.7	388.0	91.3	1.00		
**Employment sector**							
Public	36.0	15.1	203.0	84.9	2.58	1.43–4.66	< 0.001
Public and private	71.0	11.2	563.0	88.8	1.91	1.10–3.33	0.01
Private	14.0	5.8	226.0	94.2	1.00		
**Adequate knowledge**							
Yes	85.0	17.2	410.0	82.2	2.94	2.03–4.27	< 0.001
No	36.0	5.8	582.0	94.2			

Abbreviations: 95%CI, 95% confidence interval; PR, prevalence ratio; SD, standard deviation.

Note: *Fisher's exact test.

With respect to practice, the mean episiotomy rate reported was 42.1% ± 29.6% (range: 0–100%), with a median rate of 40%, and frequency distribution as follows: zero (2.4%); < 20% (31.5%); 20% to 40% (21.9%); 40% to 60% (21.4%); and > 60% (22.9%). Adequate practice was associated with: age < 40 years (PR: 1.8; 95%CI: 1.5–2.2); being female (1.2; 0.9–1.4); accessing healthcare databases (1.4; 0.9–2.2); teaching (1.5; 1.2–1.8); time since graduation ≤ 10 years (2.0; 1.6–2.7); working in a state capital city (1.3; 1.1–1.7); and working in the public sector (2.3; 1.7–3.3). It was also associated with having adequate knowledge (2.7; 2.22–3.41) and adequate attitude (3.0; 2.57–3.63) (Table 3).

**Table 3:** Factors associated with adequate practice of Brazilian obstetricians regarding episiotomy

Variables	Adequate		Inadequate		PR	95%CI	*p*-value
	N	%	N	%			
**Age (mean/SD)**	44.1	11.8	44.9	12.1			0.7
**Age < 40 years**							
Yes	178.0	35.7	320.0	64.2	1.83	1.50–2.23	< 0.001
No	120.0	19.5	495.0	80.5	1.00		
**Gender**							
Female	174.0	29.1	423.0	70.9	1.21	0.99–1.47	0.02
Male	124.0	24.0	392.0	76.0	1.00		
**Participation in congresses**							
Yes	289.0	26.6	796.0	73.4	0.82	0.47–1.43	0.25
No	9.0	32.1	19.0	67.9	1.00		
**Access to databases**							
Yes	280.0	27.5	738.0	72.5	1.45	0.94–2.22	0.03
No	18.0	18.9	77.0	81.1	1.00		
**Teaching**							
Yes	166.0	33.1	335.0	66.9	1.53	1.26–1.86	< 0.001
No	132.0	21.6	480.0	78.4	1.00		
**Time since graduation**							
≤ 10 years	137.0	36.0	244.0	64.0	2.09	1.60–2.73	< 0.001
10–25 years	101.0	26.4	282.0	73.6	1.53	1.15–2.03	0.002
> 25 years	60.0	17.2	289.0	82.8	1.00		
**Highest degree**							
Specialist	200.0	25.8	574.0	74.2	0.88	0.59–1.31	0.54
Master's degree	49.0	30.2	113.0	69.8	1.03	0.66–1.61	0.87
Doctor of Philosophy (PhD)	30.0	26.8	82.0	73.2	0.91	0.56–1.49	0.72
Doctor of Medicine (MD)	19.0	29.2	46.0	70.8	1.00		
**Place of work**							
State capital city	170.0	28.9	418	71.1	1.39	1.11–1.74	0.003
State capital and other cities	40.0	40.0	60.0	60.0	1.93	1.42–2.61	< 0.001
Outside state capital city	88.0	20.7	337.0	79.3	1.00		
**Employment sector**							
Public	88.0	36.8	151.0	63.2	2.38	1.70–3.35	< 0.001
Public and private	173.0	27.3	461.0	72.7	1.77	1.28–2.44	< 0.001
Private	37.0	15.4	203.0	84.6	1.00		
**Adequate knowledge**							
Yes	205.0	41.4	290.0	58.6	2.75	2.22–3.41	< 0.001
No	93.0	15.0	525.0	85.0			
**Adequate attitude**							
Yes	81.0	66.9	40.0	33.1	3.06	2.57–3.63	< 0.001
No	217	21.9	775.0	78.1			

Abbreviations: 95%CI, 95% confidence interval; PR, prevalence ratio; SD, standard deviation.

In the multivariate analysis, the factors that remained associated with adequate knowledge were: age < 40 years (odds ratio [OR]: 1.5; 95%CI: 1.1–1.9); teaching (1.6; 1.3–2.1); and working in the public sector (1.5; 1.1–2.1). Adequate attitude remained associated with: adequate knowledge (3.0; 2.0–4.6); age < 40 years (2.0; 1.3–3.1); teaching (1.6; 1.0–2.4); and being female (1.0; 1.0–2.4). Adequate practice remained associated with: adequate knowledge (3.1; 2.3–4.2); adequate attitude (5.0; 3.2–7.7); age < 40 years (1.9; 1.4–2.6); working in a state capital city (1.5; 1.0–2.0); teaching (1.5; 1.1–2.0); and working in the public sector (1.6; 1.0–2.5) (Table 4).

**Table 4:** Multivariate analysis: factors associated with adequate knowledge, attitude and practice of Brazilian obstetricians regarding episiotomy

Variable	OR	95%CI	Coefficient	Standard error	Z	*p*-value
*Adequate knowledge*						
Age < 40 years	1.51	1.18–1.93	0.41	0.12	3.32	< 0.001
Teaching	1.67	1.30–2.13	0.51	0.12	4.07	< 0.001
Working in the public sector	1.57	1.15–2.15	0.45	0.15	2.89	0.003
Constant	*	**	-1.00	0.15	-6.61	< 0.001
*Adequate attitude*						
Adequate knowledge	3.04	2.00–4.62	1.11	0.21	5.21	< 0.001
Age < 40 years	2.03	1.32–3.13	0.71	0.21	3.25	0.001
Teaching	1.62	1.08–2.42	0.48	0.20	2.37	0.017
Being female	1.58	1.02–2.45	0.46	0.22	2.07	0.038
Attending congresses	0.32	0.11–0.87	-1.12	0.50	-2.22	0.026
Constant	*	*	-2.53	0.53	-4.77	< 0.001
*Adequate practice*						
Adequate knowledge	3.12	2.30–4.22	1.13	0.15	7.39	< 0.001
Adequate attitude	5.02	3.26–7.73	1.61	0.22	7.32	< 0.001
Age < 40 years	1.97	1.45–2.66	0.68	0.15	4.41	< 0.001
Working in a state capital city	1.50	1.09–2.06	0.40	0.16	2.50	0.012
Teaching	1.50	1.11–2.04	0.41	0.15	2.63	0.008
Working in the public sector	1.65	1.09–2.50	0.50	0.21	2.37	0.017
Constant	*	*	-3.03	0.25	-11.96	< 0.001

Abbreviations: 95%CI, 95% confidence interval; OR, odds ratio.

High episiotomy rates, above 20%, were found among more than 60% of the physicians who reported performing the procedure “always,” “sometimes” or “seldom”, and among 40% of those who stated that they “never” performed the procedure. Nevertheless, practice was considered adequate in a significantly higher number of the respondents who reported never performing the procedure (60%) (Table 5).

**Table 5:** Association between practice reported by the participants and adequate practice as defined by the authors

Variables	Adequate		Inadequate		Chi-squared	*p*-value
	N	%	N	%		
***Performance of episiotomy***						
Always*	43.0	33.6	85.0	66.4	12.61	0.005
Sometimes**	215.0	31.3	472.0	68.7		
Seldom***	100.0	37.3	168.0	62.7		
Never****	18.0	60.0	12.0	40.0		

Notes: Frequency reported by the participant: *median: 45; range: 0–100; interquartile range (IQR): 20–80; **median: 40; range: 0–100; IQR: 20–60; ***median: 40; range: 0–100; IQR: 15–60; ****median: 20; range: 0–90; IQR: 5–40.

## Discussion

The present survey indicates a considerable gap in the knowledge, attitude and practice of Brazilian obstetricians in relation to the recommendations and scientific evidence on episiotomy. Considering the Cochrane systematic review,^1^ the ACOG^4^ and the WHO guidelines^3^ as the principal sources of data with respect to knowledge on episiotomy, the rate of adequate knowledge was found to be of 45.5%. This rate is indicative of a failure in the transfer of currently existing knowledge on the procedure.

Considering the time and the volume of publications on the subject, including the Brazilian Ministry of Health guidelines, from 2000,^19^ with a new publication in 2017 (Brazilian Guidelines to Normal Birth)^20^ and those of the FEBRASGO itself,^21^ this finding is a concern. In accordance with the conclusions reached in the Cochrane systematic review, FEBRASGO's most recent manual on healthcare at childbirth^22^ emphasizes the benefits of restricting the practice of episiotomy.^1^

Among the factors more commonly associated with adequate knowledge, both age and time since graduation are indicative of younger individuals whose medical training took place under a new paradigm. In addition, it is more likely that these younger doctors had access to more recent scientific evidence on the subject during their undergraduate courses or residency programs. On the other hand, having a postgraduate degree may improve access to health databases, conferring greater familiarity with the concepts of evidence-based medicine and a greater capacity to comprehend the evidence.

Working in a state capital city and in the public sector also facilitates compliance with more contemporary protocols, bearing in mind that most medical residency programs are in the public sector, and, therefore, involve clinical meetings and their own manuals and routines. Individuals who work exclusively in smaller towns and rural areas may find it more difficult to keep updated. Teaching also suggests that doctors are more concerned with research and with studying scientific evidence to enable them to provide their students with adequate knowledge. In the multivariate analysis, the variables that remained significantly associated with adequate knowledge were: age < 40 years; teaching; and working in the public sector.

One important question is whether knowledge is transferred to practice. Although an association was found between knowledge and attitude and practice in relation to episiotomy, attitude and practice were more often inadequate compared with knowledge, and the same individuals whose knowledge was adequate were often found to have inadequate attitudes and practices.

A study conducted in the United Kingdom^23^ evaluated the importance of accessing databases on the practices of obstetricians and midwives. Access was defined as visiting Cochrane systematic review internet pages. The authors found that access to scientific evidence was insufficient to change professional practice.^23^

Studies have shown the benefits of investigating and being aware of the best scientific evidence, since obtaining new knowledge is the first step toward changing a practice. Nevertheless, there has always been a gap between scientific evidence (what is known) and the clinical practice (what is done). The reasons for this gap are complex; however, the difficulty in establishing a bridge between evidence and clinical practice is clear.^24^

The results of a KAP study^25^ on episiotomy conducted with Vietnamese-born women in Australia and involving doctors and midwives were merely descriptive, and the associated factors were not evaluated, thus hampering comparison with the present study. In that study, however, the authors concluded that obstetricians and midwives in Vietnam held erroneous beliefs regarding the reasons to perform episiotomy and its consequences, contradicting current evidence. The majority of those professionals reported performing episiotomy in around 90% of nulliparous women.^25^

In relation to attitude, most of the answers in the present survey were classified as inadequate (89.1%). It is noteworthy that even among professionals with adequate knowledge, only 17% had attitudes that were considered adequate. A new element involving an issue of gender appears here, since adequate attitude was more prevalent among women, who may have been more careful about preserving perineal integrity and more concerned with the harmful effects of episiotomy than men. In the multivariate analysis, the factors that remained significantly associated with adequate attitude were: adequate knowledge; being < 40 years of age; teaching; being female; and attending congresses.

Recently, a cross-sectional study^26^ was conducted with midwives in the Kurdistan Region of Iraq on those professionals' perception of the measures adopted to prevent second-degree lacerations of the perineum. The majority of the professionals were of the opinion that episiotomy should be performed routinely in the hospitals in that country, an opinion comparable to the inadequate attitude of the professionals in the present study. Furthermore, in agreement with the present results, the knowledge of those professionals was weak in relation to the consequences of episiotomy for the perineum.^26^

Another study^27^ compared the attitudes of obstetricians over 40 years of age working in Canadian hospitals with those of their younger colleagues. The younger obstetricians were found to have a better opinion in relation to scientific evidence compared with the older doctors. In general, the younger doctors were more careful with respect to the guidelines on the use of technology in normal childbirth.^27^ The results of the present study also showed that being younger and having graduated more recently were factors that contributed positively toward having adequate attitude.

Inadequate practice predominated in most of our sample. The mean frequency of episiotomy reported was of 42%. As an ideal rate of episiotomy is not established, we used a cut-off point of 20%, trying to include a larger number of obstetricians and facilitate analysis, since only 30 (2.7%) stated that they never performed the procedure.

In the Cochrane systematic review, the episiotomy rate described in the selective episiotomy group ranged from 8%^28^ to 59%^29^ in the different studies. The rate was of 10% in a large randomized controlled trial (RCT) conducted in the United Kingdom,^30^ and of 30% in a large Argentinian RCT.^31^ The overall frequency rate was of 28.4% versus 75% in the routine episiotomy group.^1^ In other words, there were considerable variation and heterogeneity in those studies. Indeed, the “ideal” episiotomy rate remains to be established, since its actual indications^1^ are unclear, and there are authors who defend the idea that the procedure should never be performed.^32^ Although it has been shown that it is possible to achieve much lower episiotomy rates,^7^ ^33^ or even a 0% rate with no adverse outcomes,^33^ we believe that a maximum rate of 20% would be more reasonable in the current context. Indeed, the practice of episiotomy appears to have become so commonplace that even among those obstetricians who stated that they never perform the procedure, 40% actually perform episiotomy in more than 20% of their deliveries.

Younger professionals and those who graduated more recently may be more open to changing their practices when confronted with evidence, or they may have been trained within a new paradigm of obstetric care. There have already been reports of medical residency programs in certain Brazilian healthcare services in which the episiotomy rate is below 2%.^33^ Working in a state capital city and in the public sector may also lead to favorable changes in practice, since these hospitals are more likely to provide training opportunities for their clinical staff, and it is in such institutes that concern is greater with the good practices recommended in the Ministry of Health's national policy to humanize medicine.^19^ As expected, having access to databases and teaching are factors that influence medical practice. Nevertheless, even among the obstetricians with adequate knowledge, the practice of episiotomy was only adequate for 41.4%, and the association between attitude and practice was stronger (among 67% of the obstetricians with adequate attitude, practice was also adequate).

Gender did not remain significant in the multivariate analysis. Despite the fact that the frequency of adequate attitude and practice was higher among women, they continued to perform the procedure at much higher rates than those recommended by the WHO, and well above those considered adequate by the authors of the present study (practice was only adequate in 29%). This may indicate a certain difficulty in abandoning old habits, with technocratic medical training taking precedence over the female doctor's self-identification with the problem of episiotomy and the ritualistic nature of the procedure.^34^

The patterns of episiotomy performed by residents and preceptors of a teaching hospital in Pennsylvania, United States, and doctors in that hospital's private clinic were evaluated.^35^ The episiotomy rate decreased from 59.7% to 45% over the study period, which was from 1995 to 2000. In agreement with the present results, the episiotomy rate was higher among the professionals in the private clinic compared with the residents and preceptors. This finding was attributed to the period during which those professionals received their training; however, the study was limited because no information was available on age, gender, years of experience in medical practice, or the amount of episiotomy training each doctor had received during their residency period.^35^

A Brazilian study^10^ evaluated good practices in the care provided at childbirth to 23,894 women at low obstetric risk from every geographical region of the country. A high frequency of episiotomy (54%) was found,^10^ similar to the results encountered in the present survey, in which the majority of the professionals reported performing episiotomy in over 40% of deliveries.

The persistence in performing episiotomy at a considerable proportion of vaginal deliveries could be explained by the guidance given in the obstetrics textbooks widely adopted throughout the country, by the academic training provided, by the experiences in teaching hospitals and, mainly, by the difficulty in changing behavior and transferring scientific evidence and clinical recommendations to the practice.^36^

In Brazil, medical training continues to be excessively technocratic, and the female body is seen as essentially defective and dependent on medical intervention to enable the birth process to occur.^37^ Despite the accumulated evidence that routine episiotomy is unnecessary, the procedure is taught and performed in many training centers in Brazil. According to American anthropologist Robbie David-Floyd, episiotomy and so many other unnecessary or harmful obstetric procedures tend to persist irrespective of their effectiveness, safety, or the suffering they may cause, because they are “rational ritual responses to our technocratic society's extreme fear of the natural processes on which it still depends for its continued existence.”^38^

The present study has limitations. Since only a proportion of Brazilian obstetricians answered the survey, its findings cannot be generalized to all Brazilian obstetricians. Nevertheless, we consider the response rate satisfactory, bearing in mind the indirect route used to contact the doctors and the inherent difficulties in obtaining answers to questionnaires sent by e-mail. We do not know how many physicians actually received the three invitations sent to them by e-mail because the addresses used may not have been current, or the messages may even have gone directly to the recipient's spam box, going unnoticed. However, the response rate is similar to that reported in another study^39^ that used a similar methodology. As strengths of the present study, we found that the tool used for data collection provided an easy way to reach an expressive number of doctors, reaching all regions of Brazil. In addition, the form was able to address the main questions about the KAP regarding episiotomy.

The physicians who failed to answer the survey may have been different from those who answered. They may have had little interest in the subject, or have been too busy or unwilling to participate in online research. However, if this bias exists, we believe that it would be toward overestimating KAP, since it appears reasonable to believe that those most interested in the subject would have been the ones willing to participate. The study also failed to assess the type of education and training on episiotomy that the professionals had during their medical training and residency, a factor that may have affected the results. Nevertheless, we believe that we have successfully dealt with this limitation, albeit indirectly, with the questions involving KAP.

These results pose new challenges, both in the clinical practice and in research. The high rates of episiotomy in Brazil need to be evaluated critically, as does the inadequacy of the KAP of Brazilian obstetricians. This includes questioning the current training process and the actual model of healthcare applied to women at childbirth in this country.

Continued education and programs aimed at sensitizing the professionals will enable episiotomy rates to be reduced. It is also important to be aware of the main factors associated with this procedure.^40^ The pertinent scientific evidence regarding episiotomy should be widely disseminated both in medical schools and residency programs, as well as in congresses and training courses.

More effective methods of transferring currently available scientific evidence to healthcare professionals should be identified, as well as more appropriate ways of guaranteeing that the knowledge acquired is applied in practice.^36^ Strategies have to be developed to sensitize doctors, enabling them to reflect on why they continue to perform episiotomies so often despite the scientific evidence against the procedure. Since episiotomy is often performed ritualistically, changing practices may require a lot more than increasing the level of knowledge on the subject. Work has to be done within what has recently been described as “quaternary prevention,”^41^ which may require profound changes in the teaching of obstetrics, beginning at the university level. Recalling the Hippocratic injunction “*Primum non nocere*” (“above all, do no harm”), it is necessary to “identify those individuals who are at risk of being hyper-medicalized and reduce unnecessary or excessive interventions in order to minimize iatrogenesis.”^42^

## Conclusion

Most participants had inadequate KAP regarding episiotomy. Although some factors such as age, teaching, working in the public sector and attending congresses improved KAP, we have to recognize that episiotomy rates remain well above what would be considered ideal. Adequate knowledge is more prevalent than adequate attitude or practice, indicating that improving knowledge is crucial but insufficient to change the outlook of episiotomies in Brazil.
